# Differences in risk perception, knowledge and protective behaviour regarding COVID-19 by education level among women and men in Germany. Results from the COVID-19 Snapshot Monitoring (COSMO) study

**DOI:** 10.1371/journal.pone.0251694

**Published:** 2021-05-12

**Authors:** Petra Rattay, Niels Michalski, Olga Maria Domanska, Anna Kaltwasser, Freia De Bock, Lothar H. Wieler, Susanne Jordan

**Affiliations:** 1 Robert Koch Institute, Berlin, Germany; 2 Federal Centre for Health Education, Cologne, Germany; University of New South Wales, AUSTRALIA

## Abstract

The main strategy for combatting SARS-CoV-2 infections in 2020 consisted of behavioural regulations including contact reduction, maintaining distance, hand hygiene, and mask wearing. COVID-19-related risk perception and knowledge may influence protective behaviour, and education could be an important determinant. The current study investigated differences by education level in risk perception, knowledge and protective behaviour regarding COVID-19 in Germany, exploring the development of the pandemic over time. The COVID-19 Snapshot Monitoring study is a repeated cross-sectional online survey conducted during the pandemic in Germany from 3 March 2020 (waves 1–28: 27,957 participants aged 18–74). Differences in risk perception, knowledge and protective behaviour according to education level (high versus low) were analysed using linear and logistic regression. Time trends were accounted for by interaction terms for education level and calendar week. Regarding protective behaviour, interaction terms were tested for all risk perception and knowledge variables with education level. The strongest associations with education level were evident for perceived and factual knowledge regarding COVID-19. Moreover, associations were found between low education level and higher perceived severity, and between low education level and lower perceived probability. Highly educated men were more worried about COVID-19 than those with low levels of education. No educational differences were observed for perceived susceptibility or fear. Higher compliance with hand washing was found in highly educated women, and higher compliance with maintaining distance was found in highly educated men. Regarding maintaining distance, the impact of perceived severity differed between education groups. In men, significant moderation effects of education level on the association between factual knowledge and all three protective behaviours were found. During the pandemic, risk perception and protective behaviour varied greatly over time. Overall, differences by education level were relatively small. For risk communication, reaching all population groups irrespective of education level is critical.

## Introduction

Since the beginning of 2020, the COVID-19 pandemic has been severely impacting global health [1]. As in many other countries, Germany has seen an increase in the number of SARS-CoV-2 infections and has attempted to reduce the spread of the COVID-19 pandemic through targeted containment measures implemented by national government and local authorities [2]. In 2020, when vaccinations were not yet available for a large proportion of the population, the response focused on non-pharmaceutical public health measures [3]. The official German containment strategy in 2020 was based on three main non-pharmaceutical measures communicated by the distance-hygiene-mask (DHM) formula [4], conveying the key directives for individual behaviour: (1) maintaining a physical distance of 1.5 meters (D), (2) following hygiene rules (H) and (3) wearing non-medical face masks (community masks) in crowded public spaces and where maintaining distance is impossible (M). Because containment measures for maintaining distance and wearing face masks were enforced in Germany for the first time, the population had to become accustomed to these new protective health behaviours.

### Theoretical background

The individual application of new forms of protective behaviour to contain COVID-19 rests on individual (informed) decisions to follow the given rules. Mainly cognitive and affective processes, but also social processes, drive the acceptance of the rules which enable information reception and influence risk perception [5]. According to Betsch [6, p.7] risk perception is “the process of constructing a subjective judgement about the risk of an option”. In the case of COVID-19, two dimensions of risk perception must be considered. First, the cognitive perception of the risk of infection by SARS-CoV-2 and of falling ill with COVID-19 are regarded as the infection probability and severity of the disease, respectively. The second dimension, affective risk perception, focuses on affective states, such as worries, fear and anxiety about being infected or getting sick. In the context of infectious diseases, risk is defined and operationalized with regard to the relevance of cognitive and affective aspects [7]. In numerous studies of previous epidemics and the current COVID-19 pandemic, risk perception is reported to be an essential factor influencing protective behaviour [8, 9].

To establish protective behaviour in the population during epidemics, risk perception is only one influencing dimension that is regarded as relevant based on psychological models of behaviour change, such as the Health Belief Model (HBM) [10], Protection Motivation Theory (PMT) [11], or motivational approaches for changing health behaviour. One further important factor is knowledge [12]. For this reason, studies of protective behaviour during epidemics also include knowledge, and have conducted knowledge-attitude-practice (KAP) surveys; in this context, attitude refers to perceived risk, and practice refers to protective behaviour [e.g., 13, 14]. Previous studies of COVID-19 protective behaviour that take knowledge into account have consistently found a positive association between knowledge level and adherence to COVID-19 protective behaviour [13, 15, 16].

For an appropriate response to the COVID-19 pandemic, media play an important role in risk communication, conveying information about the virus, contamination and treatment, as well as protective behaviour and applicable rules. Protective behaviour in the population is thought to be more in line with media coverage than the actual spread of an epidemic (“Pandemic Public Health Paradox“) [17]. In addition, studies of previous epidemics and the COVID-19 pandemic have revealed that media coverage can substantially influence public risk perception [18, 19] and protective behaviour [18, 20]. However, in the current COVID-19 pandemic, the role of perceived adequacy of media coverage has not yet been analysed in detail in the context of KAP surveys. Faasse and Newby reported results indicating that closely following media coverage is a predictor of protective behaviour [20].

Furthermore, the COVID-19 pandemic has demonstrated that the spread and burden of infectious diseases, as well as the side effects of containment measures, are unevenly distributed along lines of social deprivation, and have the potential to further increase socially-related health inequalities [21]. Social determinants, such as education, as well as gender and age, have also been either neglected or minimally considered in psychological models of behaviour change [10–12], even though behaviour-based prevention measures frequently struggle to reach population groups with the greatest need, further increasing health inequalities (prevention dilemma) [22]. Studies of the COVID-19 pandemic that consider education may therefore also provide insights for the further development of models to explain protective behaviour.

### State of research regarding differences by education level

Meanwhile, many previous studies have analysed knowledge, risk perception, and protective behaviour regarding COVID-19 during the pandemic. However, in many of these studies, differences according to the education level of respondents are neglected [23–36]. In some studies, educational differences have been reported.

Regarding knowledge about COVID-19, several studies have reported a positive association with education level [13, 14, 37–42]. However, in terms of risk perception, the findings of previous studies on differences by education level have been inconsistent. Some studies reported no differences in risk perception by education level [43–46]. In addition, although some studies found higher levels of perceived severity in study participants with little or no educational qualifications compared with highly educated individuals [47, 48], other studies reported that people with lower levels of education more often considered themselves to be at a lower risk of becoming infected with COVID-19 [37, 48]. Regarding protective behaviour against COVID-19, several studies found no differences by education level [20, 44, 49–51]. In contrast, other studies reported that individuals with lower levels of education were less likely to adhere to COVID-19 safety recommendations [39–41, 52]. Furthermore, some studies reported different patterns of associations with education level for various protective behaviours [37, 53]. Ciancio et al. [48] tested for interaction effects between education level and age, and found that educational differences were evident only among older people, with college-educated older individuals practicing social distancing significantly more often than non-college-educated older individuals.

To date, only a few studies have explicitly focused on education-related differences in risk perception, knowledge and protective behaviour regarding COVID-19. Findings have been relatively ambiguous, and only comparable to a limited extent. Moreover, differences between women and men in the associations between education and risk perception, knowledge and protective behaviour have not yet been examined in sufficient depth. Almost all previous studies in this area only covered the early months of the pandemic in 2020, and have tended to examine relatively small samples. Associations may also vary from country to country, depending on the educational system, the risk communication strategy, and the imposed protective measures [45]. Therefore, it must be acknowledged that results from other–especially non-Western–countries cannot simply be transferred to Germany. In addition, the course of the pandemic and protection measures, which are often adjusted, could be expected to influence risk perception, knowledge and protective behaviours [54, 55].

### The German context: The development of SARS-CoV-2 infections in 2020

**Fig 1:**
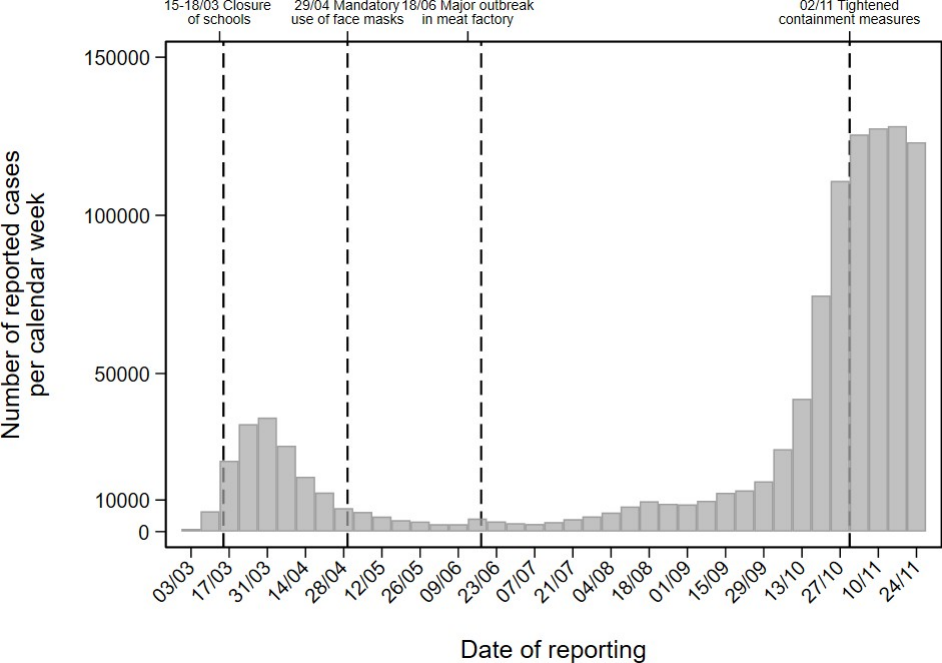
Number of COVID-19 cases reported to the Robert Koch Institute according to the onset of the disease, alternatively according to the reporting date [56].

In Germany, from the beginning of the pandemic in March until September, the numbers of SARS-CoV-2 infections have been considerably smaller compared with most other Western European countries (cf. Fig 1). By 24 November 2020, 942,687 infections (cumulative) with SARS-CoV-2 and 14,361 deaths due to COVID-19 were reported in Germany in total [56]. The first death due to COVID-19 in Germany was recorded on 09 March 2020. From the beginning of March, the numbers of new infections increased substantially. In mid-March, childcare centres, schools, restaurants and stores were closed (except for daily needs). On 23 March 2020, the federal government and federal states implemented comprehensive restrictions of social contacts. Meetings of more than two persons were prohibited, with the exception of persons living in the same household. In public, a minimum distance of 1.5 meters had to be maintained. After the rate of new infections slowly dropped over the course from mid-March to late April, the contact restrictions were released at the end of April. However, the obligation to wear a mask to cover mouth and nose in certain places and situations (e.g., in stores, public transport, and public buildings) was permanently introduced in all federal states, accompanied by the DHM formula. After relatively few new SARS-CoV-2 infections from May to September 2020, an increase in infections has been recorded since October 2020. Due to an accelerated increase of transmissions in October 2020 with a nationwide 7-day incidence of 110.9 cases per 100,000 population on 31 October, the federal and state governments agreed to introduce tightened regulations on gatherings and mask wearing, effective from 2 November 2020, initially until the end of November. As daily incidence did not drop considerably, these measures have been extended. On 16 December, stricter restrictions of social contacts came into force again. Childcare centres, schools, and stores (except for daily needs) were closed once more. These restrictions have been imposed to reduce the 7-day incidence to a moderate number of 50 cases per 100,000 people in the population.

### Aim of the study and research questions

As described above, the role of education has only partially been considered in psychological models of behaviour change. Furthermore, previous findings regarding differences by education level in risk perception, perceived media coverage, knowledge and protective behaviour have been heterogeneous. The course of the COVID-19 pandemic and the timing of protection measures might be expected to have an effect on the association between education level and risk perception, knowledge and protective behaviours. However, this aspect has not yet been investigated in detail.

Against this background, the present study was conducted to explore education-related differences in risk perception, knowledge and protective behaviour regarding COVID-19 among women and men in Germany over the course of the pandemic. We addressed the following research questions in detail:

1. Are there differences in risk perception, perceived media coverage, knowledge and protective behaviour in women and men according to education level?
2. Have the differences in risk perception, perceived media coverage, knowledge and protective behaviour according to education level changed over the course of the COVID-19 pandemic?
3. Does education level impact adherence to protective behaviour in women and men when considering risk perception, perceived media coverage and knowledge?
4. Is the impact of risk perception, perceived media coverage and knowledge on protective behaviour in women and men moderated by education level?

## Materials and methods

### Data

Analyses were conducted using data gathered by the COVID-19 Snapshot Monitoring (COSMO) study [57, 58]. The COSMO study is based on a weekly repeated cross-sectional online survey monitoring knowledge, risk perception, protective behaviour and trust over the course of the COVID-19 pandemic in Germany. COSMO is a joint project of the University of Erfurt, the Robert Koch Institute, the Federal Centre for Health Education, the Leibniz Centre for Psychological Information and Documentation, the Science Media Center, the Bernhard Nocht Institute for Tropical Medicine, and the Yale Institute for Global Health.

The study participants are part of an online access panel provided by Respondi (https://www.respondi.com/). The first survey started on 3 March 2020 and was repeated on a weekly basis. From 26 May 2020 to 27 October 2020 the time interval between waves was extended to 2 weeks, and set back to weekly afterwards. At the end of November 2020, data from 28 waves were available for data analysis.

For each survey wave, a sample of 1,000 participants aged 18–74 years was targeted. In the present analyses, data from waves 1 to 28 were used (N[min] = 925, N[max] = 1032). The total sample size is 27,957 (51% women, 49% men). Until wave 19, the cross-sectional sampling was restricted to one-time participation. This restriction was released from wave 20 onwards. In general, case numbers of repeat respondents remain low (N<9) with the exception of waves 20 to 22, in which approximately 575 of the respondents were sampled from participants who already participated in earlier waves. Repeated participation was controlled for in the multivariate analyses. A detailed description of the sample is presented in Table 1.

**Table 1:** Sample description.

Outcome variables			
	Mean	n	Missing %
Perceived susceptibility (1–7)	3.86	27,957	0.00
Perceived probability (1–7)	3.63	27,777	0.64
Perceived severity (1–7)	4.12	27,957	0.00
Worries (1–7)	4.76	27,957	0.00
Fear (1–7)	4.24	27,957	0.00
Perceived media coverage (1–7)	3.40	27,957	0.00
Perceived knowledge (1–7)	4.90	27,957	0.00
	Proportion (%)	n	Missing %
Factual knowledge (correct in %)	84.27	22,699	0.00
Hand washing (yes in %) (wave 2–28)	84.70	22,569	1.24
Distance maintaining (yes in %) (wave 3–28)	87.74	21,628	1.43
Mask wearing (yes in %) (wave 1–28)	67.04	18,342	2.15
**Predictor variable**			
Education level		Total: 27,957	0.00
Low	44.40	12413	
High	55.60	15544	
**Control or moderator variables**			
Gender		Total: 27,957	0.00
Female	50.55	14,133	
Male	49.45	13,824	
Age groups		Total: 27,957	0.00
18–29	19.10	5,340	
30–45	30.12	8,422	
46–60	28.01	7,831	
>60	22.76	6,364	
Community size		Total: 27,957	0.00
≤ 5,000 inhabitants	16.27	4,549	
5,001–20,000 inhabitants	21.62	6,044	
20,001–100,000 inhabitants	25.07	7,008	
100,001–500,000 inhabitants	17.77	4,967	
> 500,000 inhabitants	19.28	5,389	
Repeat respondent		Total: 27,957	0.00
No	93.74	26,206	
Yes	6.26	1,751	

The study design aims to achieve a representative distribution of respondents with respect to basic sociodemographic characteristics. The distribution of age and sex (crossed) as well as federal state (uncrossed) corresponds in the sample to the German population from German census data [59, 60].

The questionnaire comprises a series of questions and instruments that were used continuously across all waves. In addition, several questions or instruments were only included for a limited period of time. Further details regarding the study design can be found in the COSMO study protocol [57] as well as on the COSMO homepage (URL: https://projekte.uni-erfurt.de/cosmo2020/cosmo-analysis.html). The questionnaires for each wave are available from https://dfncloud.uni-erfurt.de/s/Cmzfw8fPRAgzEpA.

The study obtained ethical clearance from the University of Erfurt’s IRB (#20200302/20200501), and all participants provided written informed consent prior to data collection.

The data from the COVID-19 Snapshot Monitoring (COSMO) are stored in the repository PsychArchives (https://doi.org/10.23668/psycharchives.2776) and are made available to scientists upon request. Requests should be submitted to the COSMO consortium by contacting Prof. Dr. Cornelia Betsch, University of Erfurt, Nordhäuser Str. 63, 99089 Erfurt, Germany (cornelia.betsch@unierfurt.de).

### Variables

#### Outcome variables

Regarding risk perception, the answers to questions of interest were recorded using a 7-point scale. In the present study, we used the following items:

- Susceptibility: “How susceptible do you consider yourself to be to infection with the novel coronavirus?” Not susceptible at all (1)–Very susceptible (7).
- Probability: “How do you estimate your probability of being infected with the novel coronavirus?” Extremely unlikely (1)–Extremely likely (7).
- Severity: “How do you estimate the severity of an infection with the novel coronavirus for yourself?” Completely harmless (1)–Extremely dangerous (7).

Regarding the affective dimensions of risk perception, the following variables were included in the analysis:

- Fear: “The novel coronavirus is for me … not frightening (1)–frightening (7)”.
- Worries: “The novel coronavirus is for me … not worrying (1)–worrying (7)”.

To measure perceived media coverage, we used a single item, which describes the degree of satisfaction with the extent and way of presenting information on COVID-19:

- Perceived adequacy of media coverage: “The novel coronavirus is for me … inflated by the media (1)–not considered enough in the media (7)”.

Perceived knowledge was assessed using the following question:

- Perceived knowledge: “How do you rate your level of knowledge about the novel coronavirus?” No knowledge at all (1)–Very much knowledge (7).

Factual knowledge was measured by a binary score composed of the two items on knowledge of COVID-19 treatment options and virus transmission. The score for “correct knowledge” was dichotomized by labelling participants who had answered both knowledge questions correctly as “correct”, while participants who had answered one or both questions incorrectly or chose “don’t know” were assigned “not correct”. For samples surveyed after 9 November 2020, when results from clinical trials of a promising vaccine candidate were announced, answers containing vaccination as treatment option were classified as “correct” answers.

Protective behaviour was measured by three items which comply with the recommended DHM formula in Germany: Hand washing, distance maintaining, and mask wearing. Hand washing (at least 20 seconds) and mask wearing were asked about with the question: “How often have you adhered to the following rules in the last week to prevent the spread of and infection with the novel coronavirus?” In waves 1 to 6, only “yes” and “no” were offered as answer categories in the questionnaire. Maintaining distance (of 1.5 meters minimum to other people in public) was asked about for the first time in wave 4, with the response categories “yes” and “no”. From wave 7 onwards, more differentiated response categories were used for the three protective behaviours in the questionnaire. To ensure the inclusion of data from all waves for the present analysis, answers were combined into one dichotomous variable, by aggregating answering categories “never/rarely/sometimes” to “no” and “frequently/always” to “yes”.

Detailed information on the validity of the used items can be found in the “Survey tool and guidance” edited by the WHO Regional Office for Europe [61]. The psychological constructs of the cognitive dimensions of risk perception are validated items adapted from Brewer et al. [62], while the validated items for affective dimensions and perceived media coverage were adapted from Bradley and Lang [63]. The items measuring protective behaviours were adapted from Steel Fisher et al. [64]. Regarding knowledge, no validated psychological items were used. The data used were based on a questionnaire in German language. The literal translation of the items into English by the authors slightly differs from the question wording of the validated scales in English.

#### Predictor variable

Regarding education level, only school-completion qualifications were surveyed, but not vocational qualifications. Beginning with wave 1, education level was recorded with the answer categories “up to 9 years of school education”, “at least 10 years (without university entrance qualification)”, and “at least 10 years (with university entrance qualification)”. For the present analysis, the school-leaving qualifications that do not qualify for university attendance (“Abitur” in German) were combined into the group “low education”. “High education” was defined by a qualification for university entrance.

#### Time variable

For the second research question we used the calendar week to track changes over time, which also allowed us to explore the impact of events like implementation of containment measures and certain prominent contagion events (outbreaks). Calendar week was used as a categorical as well as a metric variable. The use of calendar week also accounts for the changing survey rhythm from weekly to bi-weekly and back.

#### Control variables

To prevent confounding of cohort, repeat respondent, urban-rural, regional effects and time effects, all analyses were adjusted for age group (“18–29 years”, “30–45 years”, “46–60 years”, “61–74 years”), repeat respondent (“yes”, “no”), (official) federal state, community size (“≤5,000 inhabitants”, “5,001–20,000 inhabitants”, “20,001–100,000 inhabitants”, “100,001–500,000 inhabitants”, “>500,000 inhabitants”), and calendar week (categorical variable). Gender (“female”, “male”) was included either as a stratification variable or as a control variable.

For the sensitivity analysis, household income was used, which was included in the questionnaire from wave 11 on, with grouped answer categories.

### Data analysis

In the first step (research question 1), we explored gender-specific educational differences for multiple indicators of risk perception and related outcomes. Differences in risk perception, perceived media coverage, knowledge and protective behaviour by education level were analysed using linear or logistic regressions (depending on the scale level of each variable) stratified by gender and adjusted for age group, repeat respondent, community size, federal state, and categorical calendar week. Because the data do not represent random samples from the German population, unadjusted means and frequencies are not informative for population inferences. However, differences between education levels within the COSMO sample provide a valid basis for evaluating educational differences in risk perception in Germany. We chose to display average adjusted predictions—predicted values for linear regressions and predicted probabilities in percent for logistic regressions [65]. They provide easily interpretable results on the scale of the outcome variables and they enable the exploration of differences between educational groups without assuming all other independent variables to be constant. Adjusted predictions for each case were calculated by inserting each case’s observed values on the independent variables into the estimated regression equation. The means of the predicted values and the average predicted probabilities were presented by each educational level alongside their respective differences and the p-value of the Wald test of the difference (Table 2).

**Table 2:** Risk perception, perceived media coverage, knowledge and protective behaviour in women and men with different education levels.

		Women				Men				Total			
		(n = 14,133)				(n = 13,824)				(n = 27,957)			
		**Education level**		**Difference**	**P-value of difference**	**Education level**		**Difference**	**P-value of difference**	**Education level**		**Difference**	**P-value of difference**
		**Low**	**High**			**Low**	**High**			**Low**	**High**		
Predicted means^1^	Susceptibility	3.91	3.86	−0.05	*0*.*082*	3.86	3.83	−0.03	*0*.*319*	3.88	3.85	−0.04	*0*.*043*
	Probability	3.56	3.71	0.14	*< 0*.*001*	3.58	3.63	0.06	*0*.*026*	3.57	3.67	0.10	*< 0*.*001*
	Severity	4.21	4.08	−0.13	*< 0*.*001*	4.14	4.07	−0.07	*0*.*009*	4.17	4.08	−0.10	*< 0*.*001*
	Worries	4.88	4.91	0.04	*0*.*242*	4.57	4.65	0.09	*0*.*004*	4.72	4.78	0.06	*0*.*003*
	Fear	4.43	4.39	−0.03	*0*.*291*	4.09	4.07	−0.02	*0*.*426*	4.26	4.23	−0.03	*0*.*176*
	Perceived media coverage	3.46	3.36	−0.10	*< 0*.*001*	3.39	3.40	0.01	*0*.*777*	3.43	3.37	−0.06	*0*.*002*
	Perceived knowledge	4.78	4.99	0.21	*< 0*.*001*	4.76	5.02	0.26	*< 0*.*001*	4.77	5.00	0.23	*< 0*.*001*
Predicted probability (%)^2^	Factual knowledge	85.2	89.3	4.0	*< 0*.*001*	81.6	86.3	4.7	*< 0*.*001*	83.4	87.9	4.50	*< 0*.*001*
	Hand washing	87.8	89.1	1.2	*0*.*035*	80.7	80.6	−0.1	*0*.*901*	84.4	84.9	0.5	*0*.*254*
	Distance maintaining	90.3	90.8	0.6	*0*.*308*	83.1	85.8	2.7	*< 0*.*001*	86.7	88.3	1.6	*< 0*.*001*
	Mask wearing	70.4	69.4	−1.0	*0*.*084*	63.6	64.6	1.0	*0*.*139*	67.0	67.0	0.0	*0*.*956*

Adjusted predictions and differences (contrasts) from linear^1^ or logistic^2^ regressions by education level (main effect), adjusted for age group, repeat respondent, community size, federal state, and calendar week.

In the second step (research question 2), educational differences in risk perception, perceived media coverage, knowledge, and protective behaviour were explored over time. We have described general trends in risk perception, knowledge and protective behaviour over the course of the pandemic and their differences by educational level. In addition, we explored whether educational differences found in the first step are stable or fluctuate over the course of the pandemic, enabling us to assess the robustness of overall differences. For this purpose, interaction terms for education level and categorical calendar week were added to the multivariate regression models and adjusted predictions were calculated for each calendar week. For descriptive purposes, we reran the same models with metric calendar week as a polynomial up to the 6th degree. Because the 6th degree polynomial function allows for up to five local extrema, it enabled us to estimate a curve that eventually corresponded to the trend in official numbers of SARS-CoV-2 infections over time, which is characterized by two major peaks and one local outbreak in the between-period. Adjusted predictions for each calendar week stratified by education from both model families were calculated and graphically displayed. The time axis displaying calendar weeks was labelled with the dates of the Tuesdays, when most of the field work was conducted in COSMO. In this step, we did not stratify the analysis by gender, but included gender as a further control variable to adjust for gender-specific intercepts.

In the third step (research question 3), for each protective behaviour (hand washing, distance maintaining, and mask wearing) logistic regressions models were estimated with education level as the main predictor, as well as all variables regarding risk perception, perceived media coverage and knowledge as further predictors (adjusted for age group, repeat respondent, community size, federal state, and calendar week).

Finally, logistic regressions for each protective behaviour (hand washing, distance maintaining, and mask wearing) were conducted including interaction terms of all risk perception variables and knowledge with education level separately. For the significant interactions between risk perception, media coverage or knowledge with education level (research question 4), we present margin plots to show the moderation effects. All models were calculated separately for women and men.

As sensitivity analyses, we added household income as a further control variable to the models for step 1 and 3 for those waves in which income data were available (from wave 11). This did not change the results reported below.

Conventional levels of significance were applied (*p < 0.05; **p < 0.01; *** p < 0.001). All analyses were performed using the Stata/SE15 statistical package (StataCorp, College Station, TX, USA).

## Results

### Differences by education level

Table 2 shows the adjusted means and frequencies for each outcome variable for the low- and high-education groups (main effects), in total and stratified by gender (research question 1).

In the total sample we found differences between individuals with low- and high-education for all three cognitive dimensions of risk perception (susceptibility, severity and probability). Low-education respondents considered themselves to be slightly more susceptible compared with high-education respondents. However, the difference in susceptibility was relatively small, and was not significant in separate gender groups. Furthermore, low-education respondents assessed SARS-CoV-2 infection as more severe. Regarding respondents’ own probability of being infected with the SARS-CoV-2 virus, the opposite association was found. Differences by education level according to severity and probability were evident in both women and men.

Concerning affective dimensions of risk perception (worries and fear), for fear, no differences by education level were observed. In terms of worries, men in the high-education group reported worries more often compared with men in the low-education group. In women, no differences by education level were found.

Media coverage of the pandemic was evaluated as insufficient more often by women in the low-education group compared with those in the high-education group, whereas, in men, no differences by education level were evident.

Regarding perceived as well as factual knowledge about COVID-19, women and men with a high level of education scored substantially higher.

In terms of protective behaviour, some differences by gender were observed. In women, hand washing was more often applied by individuals in the high-education group, whereas in men, no differences by education level were found. In contrast, for maintaining distance, higher compliance was evident for men in the high-education group compared with those in the low-education group. No significant education-related differences were found in women. In mask wearing, there were no differences by education level.

### Time-based trends of differences by education level

**Fig 2:**
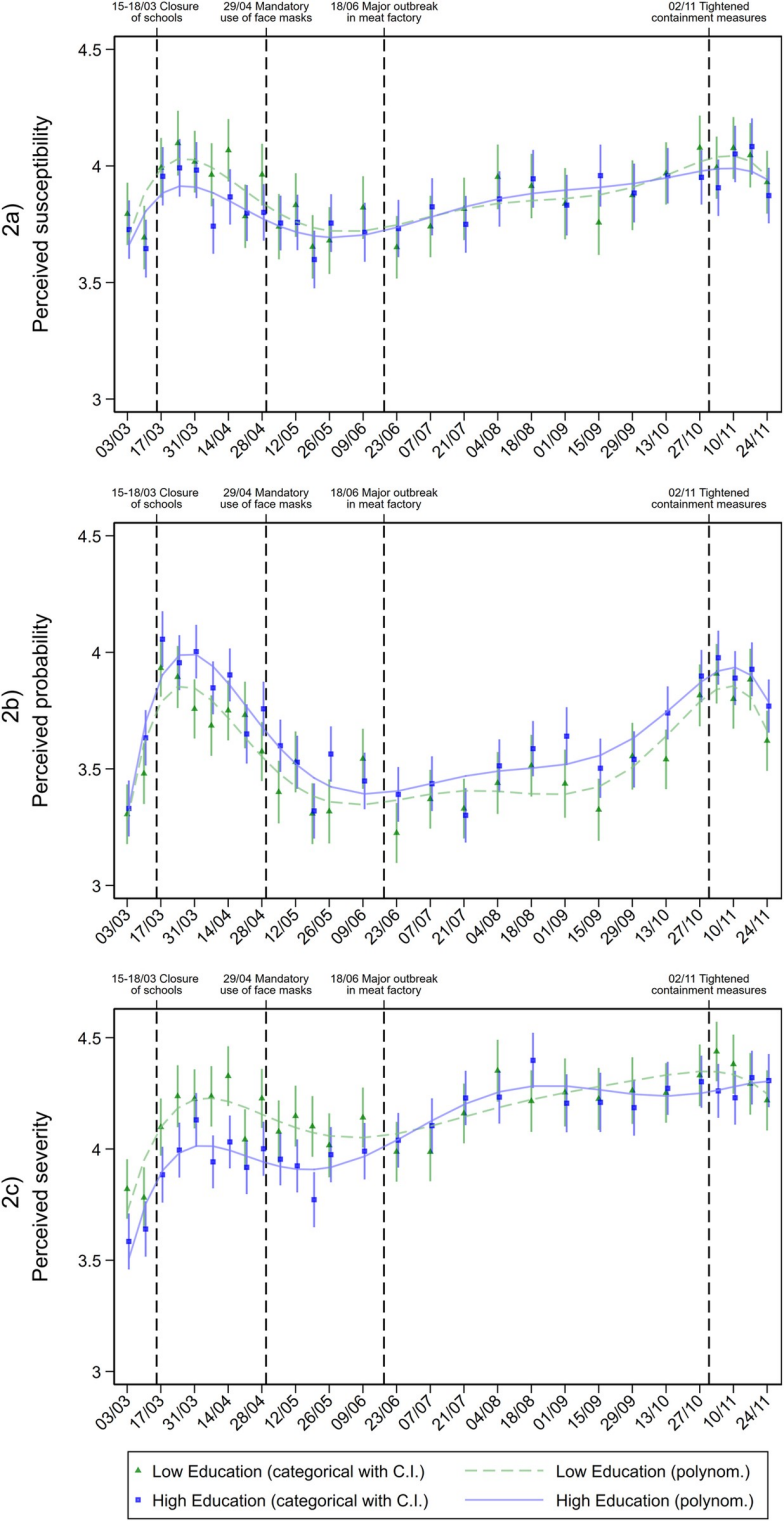
Cognitive dimensions of risk perception in individuals with different education levels during the pandemic. Adjusted predictions from linear regressions with interaction between education level and calendar week (categorial and metric), adjusted for age group, gender, repeat respondent, community size, and federal state.

**Fig 3:**
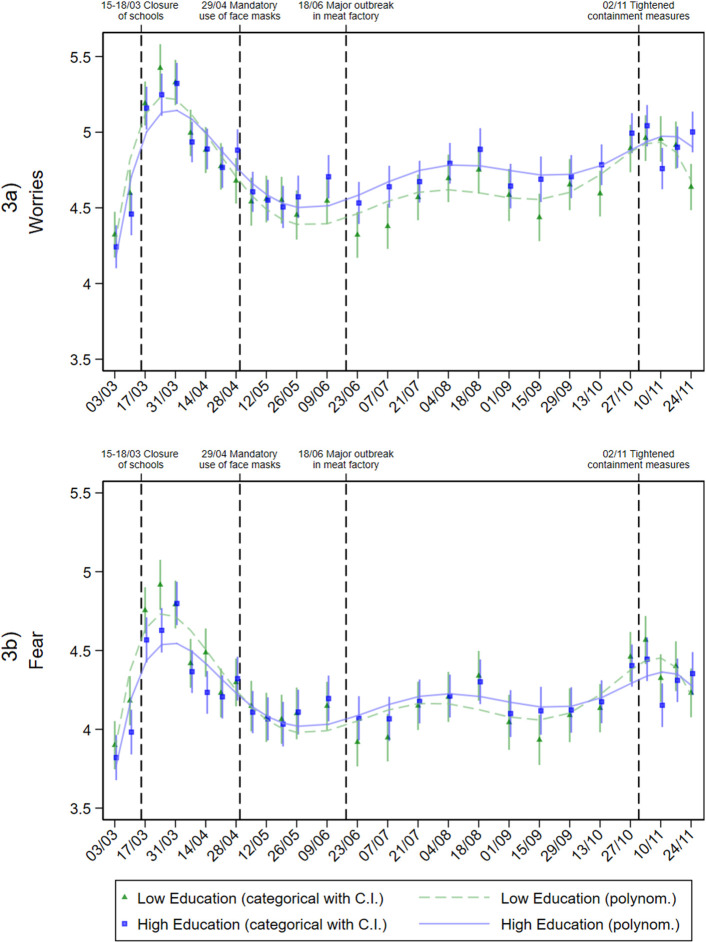
Affective dimensions of risk perception in individuals with different education levels during the pandemic. Adjusted predictions from linear regressions with interaction between education level and calendar week (categorial and metric), adjusted for age group, gender, repeat respondent, community size, and federal state.

**Fig 4:**
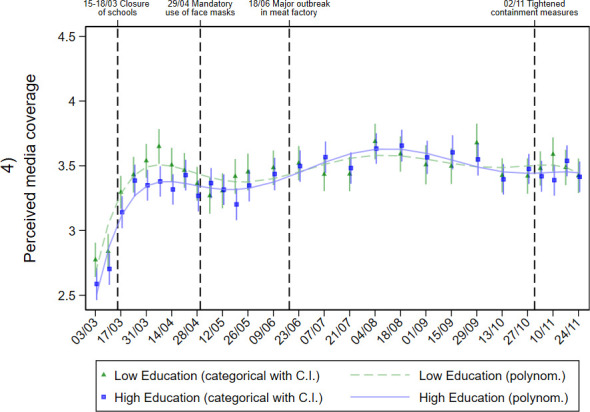
Perceived adequacy of media coverage in individuals with different education levels during the pandemic. Adjusted predictions from linear regressions with interaction between education level and calendar week (categorial and metric), adjusted for age group, gender, repeat respondent, community size, and federal state.

**Fig 5:**
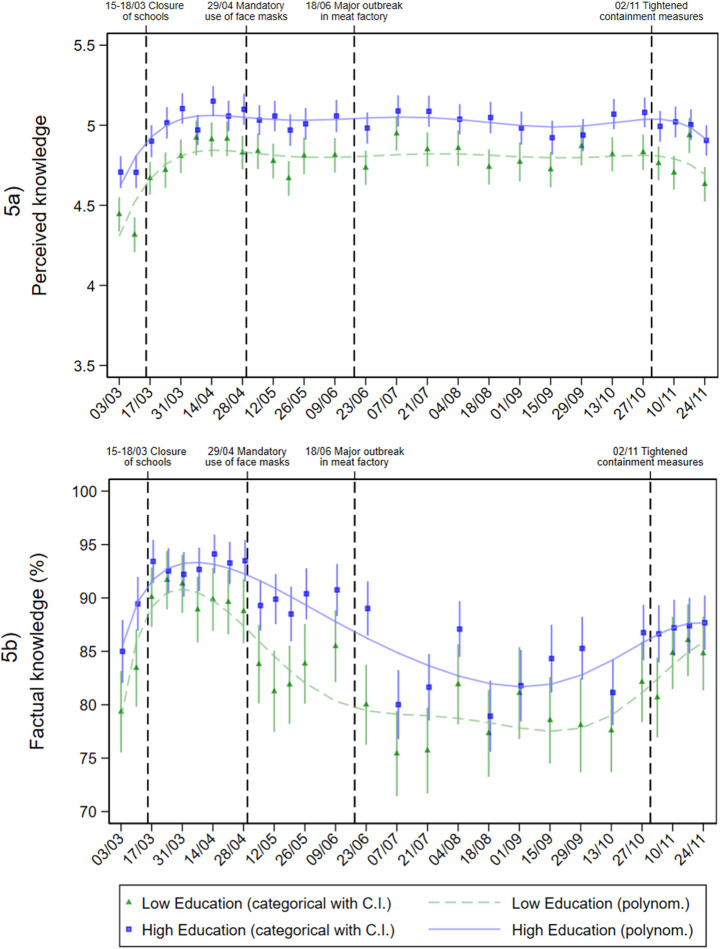
Perceived and factual knowledge in individuals with different education levels during the pandemic. Adjusted predictions from linear regression (perceived knowledge) and logistic regression (factual knowledge) with interaction between education level and calendar week (categorial and metric), adjusted for age group, gender, repeat respondent, community size, and federal state.

**Fig 6:**
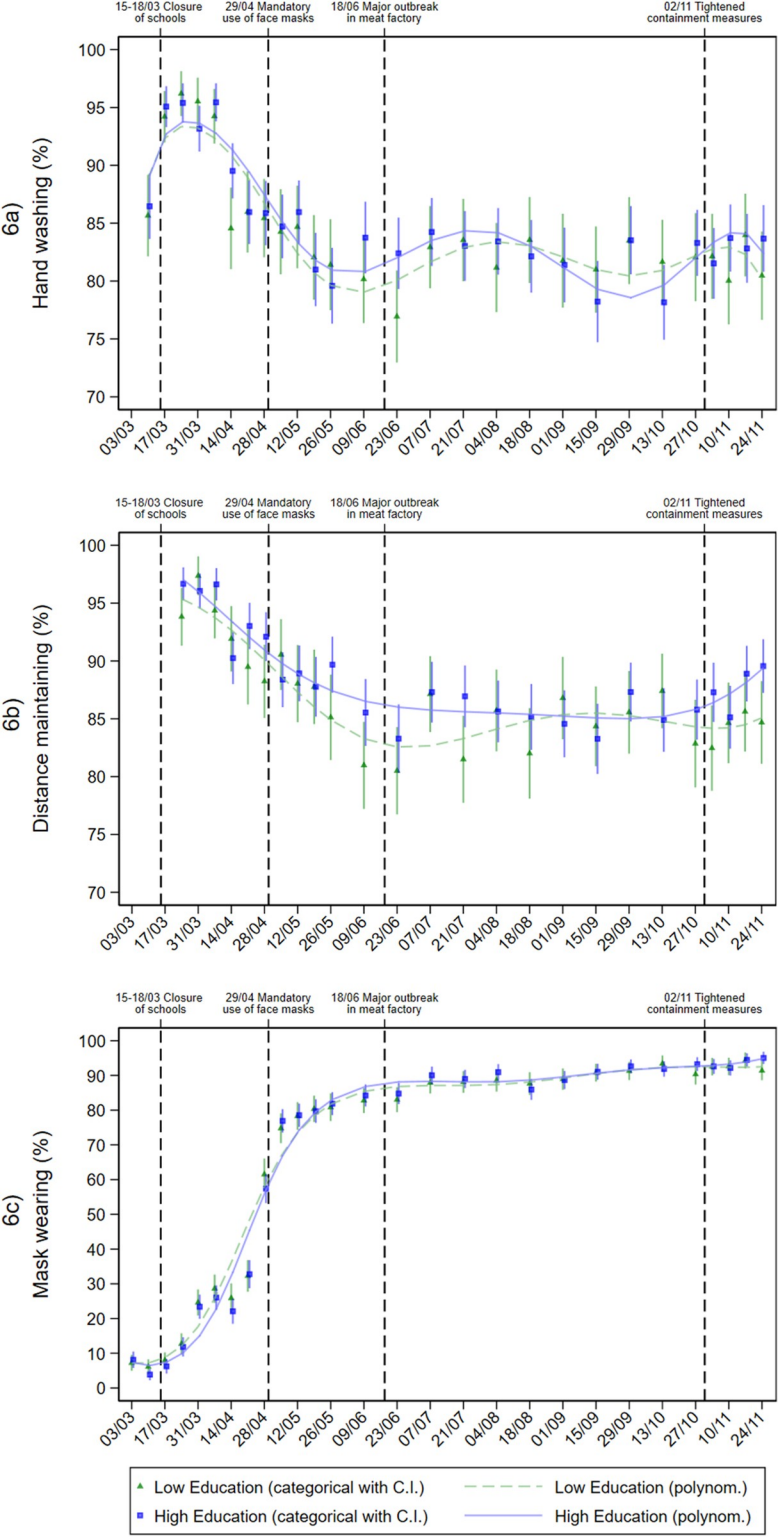
Protective behaviours in individuals with different education levels during the pandemic. Adjusted predictions from logistic regressions with interaction between education level and calendar week (categorial and metric), adjusted for age group, gender, repeat respondent, community size, and federal state.

To answer research question 2, we explored differences in risk perception, knowledge and protective behaviour by education level over time. The adjusted predictions are shown in graphical form in Figs 2–6.

The depicted growth curves for the different dimensions of risk perception principally corresponded to the time-based trends of new infections in Germany (cf. Fig 1).

For susceptibility (Fig 2A), we found small differences by education level at the beginning of the pandemic, with the low-education group considering themselves to be more susceptible to infection than the high-education group. As the pandemic progressed, education level groups converged. The trends for severity (Fig 2C) were similar to susceptibility with a more pronounced difference in the first phase. In contrast to the assessment of severity and one’s own susceptibility, the high-education group reported a higher probability of a personal infection with COVID-19. This slight difference between people with high and low education levels was consistently observed over the course of the pandemic (Fig 2B).

According to the affective dimensions of risk perception (worries and fear), only small differences by education level were observed. Nevertheless, in terms of worries (Fig 3A), a noticeable difference by education level was found for the low incidence phase from June to mid-September 2020 –unlike the cognitive dimensions of risk perception. In these months, individuals with higher levels of education consistently reported higher levels of worries. In terms of fear (Fig 3B), a trend similar to the other dimensions of risk perception was observed, but no significant differences by education level were evident.

In terms of perceived media coverage (Fig 4), an increase in agreement with the statement that the SARS-CoV-2 virus is not considered enough in the media was observed during March. Until mid-April, this perception was more pronounced among low-education individuals. Since June, assessment of media coverage remained relatively constant. From this date on, no substantial differences in assessment between high- and low-education individuals were detected.

For both perceived and factual knowledge, throughout the entire course of the pandemic, women and men in the high-education group showed a higher level of knowledge compared with those in the low-education group. The average perceived knowledge level (Fig 5A) increased at the beginning of the pandemic and remained at roughly the same level from April onwards. Factual knowledge (Fig 5B) also exhibited a significant increase at the beginning of the pandemic. However, from May onwards, a significant drop in factual knowledge was observed. Since the end of October, factual knowledge increased again, particularly in the low-education group.

Fig 6 shows the trend for practicing protective behaviours over the course of the pandemic. Regarding hand washing (Fig 6A), no considerable differences by education level were observed over time. Notably, adherence rates were highest at the beginning of the pandemic, then decreased. Maintaining distance (Fig 6B) was practiced by more than 90% of the respondents in March and April almost regardless of their education level. After the first pandemic peak, compliance somewhat eased. The decline in compliance was slightly more pronounced among the low-education group, but differences levelled from August on. Regarding mask wearing (Fig 6C), a steep increase was observed over time. Readiness to wear a mask started to increase during the first lockdown period in March and April, to approximately 30%. The introduction of mandatory mask wearing at the end of April induced a widespread application of the protective behaviour to approximately 80%. From this date onwards, the proportion remained close to this level. No differences by education level were evident.

### Full adjusted effect of education level on protective behaviour

Table 3 presents the full models for all three recommended behaviours stratified by gender (main effects). Risk perception, perceived media coverage and knowledge together with education level were used as predictors for the protective behaviour (research question 3). In these final models, a high level of education was associated with lower compliance with wearing a mask among women and lower compliance with hand washing among men. Education level had no impact on maintaining distance if risk perception, perceived media coverage and knowledge were also considered, whereas, in the bivariate model, education-related differences in maintaining distance were observed among men (Table 2).

**Table 3:** Protective behaviours in women and men. Association with education level, risk perception and knowledge.

	Women			Men		
	Hand washing	Distance maintaining	Mask wearing	Hand washing	Distance maintaining	Mask wearing
Education level						
Low	ref.	ref.	ref.	ref.	ref.	ref.
High	1.04	0.96	0.87*	0.90*	1.11	1.00
Perceived susceptibility	0.97	0.97	1.01	0.99	0.98	0.99
Perceived probability	1.03	0.95	1.05	1.01	0.97	1.03
Perceived severity	1.17***	1.13***	1.15***	1.14***	1.19***	1.20***
Worries	1.14***	1.26***	1.24***	1.14***	1.29***	1.23***
Fear	1.05*	1.04	1.07*	1.03	0.96	0.98
Perceived media coverage	1.00	1.14***	1.13***	1.04*	1.11***	1.16***
Perceived knowledge	1.18***	1.13***	1.07**	1.22***	1.18***	1.10***
Factual knowledge						
Not correct	ref.	ref.	ref.	ref.	ref.	ref.
Correct	2.05***	2.47***	1.83***	2.09***	2.44***	1.89***
*N*	13,430	12,395	13,775	13,037	12,077	13,425
pseudo *R*^2^	0.10	0.15	0.51	0.11	0.16	0.38

Odds ratios (unstandardized exponentiated coefficients) from logistic regressions, adjusted for age group, repeat respondent, community size, and federal state, calendar week, repeated participant

* p < 0.05,

** p < 0.01,

*** p < 0.001.

### The moderating effect of education level

**Fig 7:**
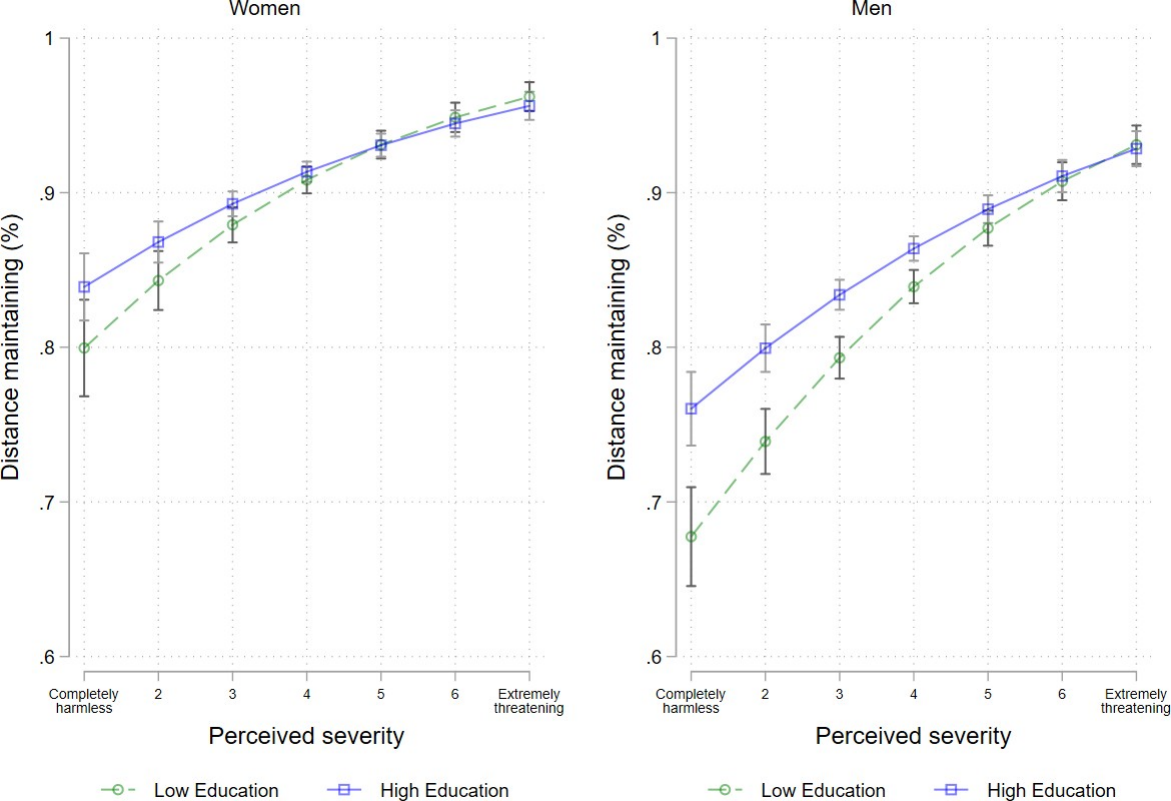
Distance maintaining by severity and education levels among women and men. Logistic regressions with interaction of severity and education level, adjusted for age group, repeat respondent, community size, and federal state, calendar week.

Regarding research question 4, we finally analysed whether the associations between risk perception, perceived media coverage or knowledge and protective behaviour were moderated by education level. Although the models for maintaining distance without interaction effects showed no significant education-related differences, we found interaction effects between education level and severity. The less severe COVID-19 was perceived to be, the greater the differences in maintaining distance by education level were (Fig 7A and 7B). Specifically, we found that, among respondents who rated the SARS-CoV-2 virus to be harmless, men in the high-education group and–to a lesser extent–women in the high-education group maintained distance more often than those in the low-education group. In contrast, no differences by education level were observed in the group who assessed the disease as severe.

**Fig 8:**
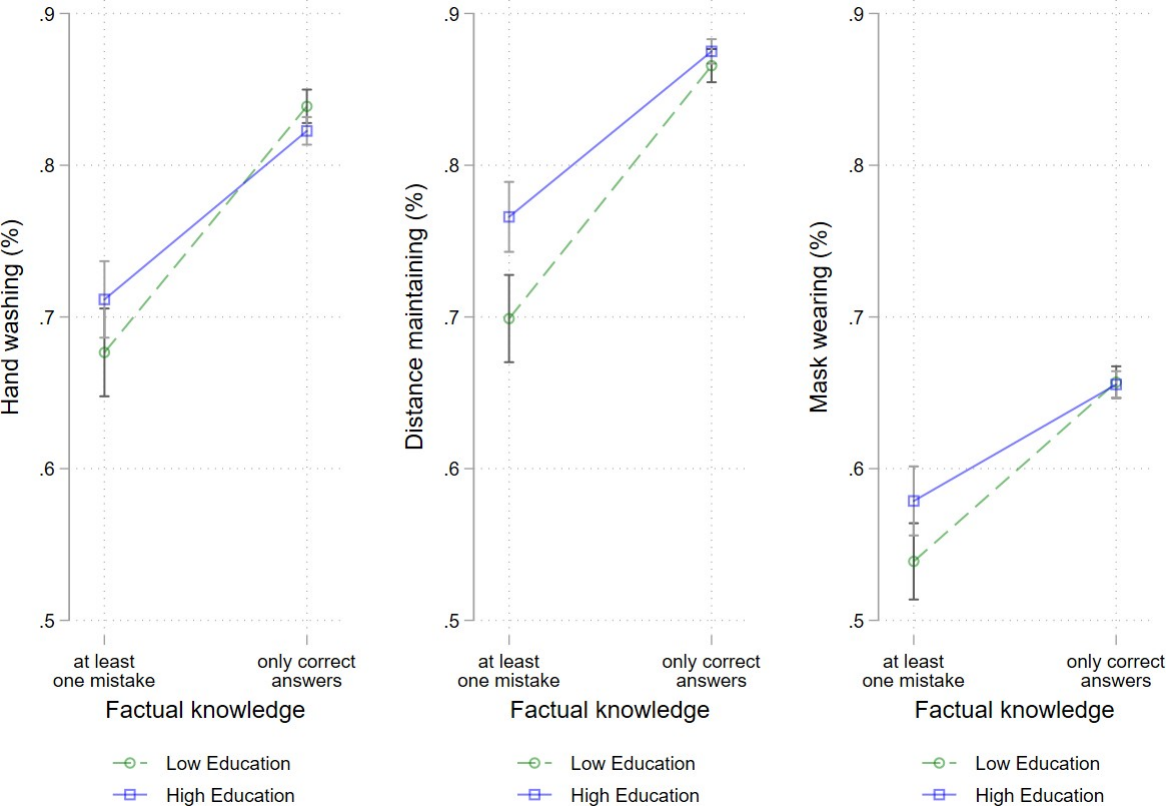
Protective behaviours by factual knowledge and education levels among men. Logistic regressions with interaction of factual knowledge and education level, adjusted for age group, repeat respondent, community size, and federal state, calendar week.

In men, the results revealed interaction effects between factual knowledge and education level with regard to all three protective behaviours (Fig 8A–8C). Thus, in men in the low-education group, differences in compliance by factual knowledge were significantly greater than those in the high-education group. The effect was most pronounced in maintaining distance. No significant moderation effect was found in women.

## Discussion

The current analysis focused on differences in risk perception, perceived media coverage, knowledge and protective behaviour regarding COVID-19 by education level during the pandemic in Germany in 2020. After a brief summary of the main findings, the results of the analysis regarding the different outcomes are discussed separately, outcome by outcome.

### Summary of findings

Analysis of differences according to education level (research question 1) revealed, that differences in knowledge were the most pronounced. The differences in risk perception, perceived media coverage, and protective behaviour were much lower, and highly heterogeneous. Thus, a lower level of education was associated with higher perceived severity, but a lower perceived probability of being infected. Regarding protective behaviour, we observed a positive association between a higher level of education and higher adherence rates for hand hygiene among women, and a positive association between a higher level of education and maintaining distance among men.

Our exploratory description of differences according to education level over the course of the COVID-19 pandemic (research question 2) revealed that differences in knowledge by education level were almost constant over time. In contrast, risk perception and protective behaviour varied markedly over the course of the pandemic, although the differences by education level were relatively small.

In the third step (research question 3) we analysed whether education level impacted adherence to protective behaviour when considering risk perception, perceived media coverage and knowledge. In the full adjusted models, we found only small effects of education level. Among those with a low level of education, the likelihood of wearing a mask was significantly higher in women, and the likelihood of hand washing was significantly higher in men.

Considering the moderating effects of education level on the association between risk perception, perceived media coverage or knowledge and protective behaviour (research question 4), we found among those who rated the SARS-CoV-2 virus to be harmless, that the high-education group maintained distance more often than the low-education group. For those who assessed the disease as severe, no differences by educational level were found. Moreover, in men, the association between factual knowledge and all three protective behaviours were also moderated by education. Thus, in the low-education group, differences in compliance by factual knowledge were significantly greater than those in the high-education group.

### Embedding the findings in the research context

We found the strongest associations of any of our considered outcomes with education level for the two indicators of personal knowledge of COVID-19 –perceived and factual, thus confirming previous research [13, 14, 37, 40–42]. Women and men with higher levels of education exhibited higher perceived as well as factual knowledge about the virus, infection and treatment. Moreover, trend analysis revealed that differences in knowledge by education level were stable over the course of the pandemic. We consider that this temporal stability also indicates a high level of reliability of the results. Salimi et al. [37] proposed that education-related differences in knowledge may be related to their finding that individuals with lower levels of education relied more on social media and less on public health resources to gain COVID-19-related information (see also [66]). The fact that misinformation about COVID-19 is widespread in social media [67, 68] could be relevant to this finding. Furthermore, a study regarding COVID-19-related health literacy in Germany revealed associations between education level and items regarding difficulties accessing information about coronavirus and protective behaviour on the internet, as well as understanding information provided by health professionals, authorities, and family members [69]. These difficulties in accessing and understanding information about COVID-19 among women and men with low levels of education are considered to be an important cause of a lower level of knowledge.

Regarding risk perception, heterogeneous patterns of associations with education level were seen: while perceived probability and severity varied according to education level among women and men, no differences by education level were found for susceptibility and fear in either women or men. In terms of worries, differences were observed only in men. The main results are in accord with findings from other studies: for perceived severity, we found an association between low levels of education and higher levels of severity, as reported by Costa et al. [47]. Moreover, our finding of an association between low education level and low perceived probability is in accord with the findings of Salimi et al. [37] and Ciancio et al. [48]. However, Salimi et al. [37] also observed an association between high education level and high susceptibility, which we did not observe in the current study. Several other studies reported no differences by education level in risk perception [43–45]. Furthermore, our trend analysis revealed that the association between different indicators of risk perception and education level varied over the course of the pandemic, supporting the assumption that differences according to education level are not stable over time, but depend on other factors, such as the infection rate, the type of risk communication and the measures imposed.

Assessment of the adequacy of media coverage showed a difference by education level only among women. Women with low levels of education more often assessed the media coverage as insufficient compared with those with a higher level of education. In another study in Germany, Okan et al. [69] found that 9 out of 10 adults felt well or very well informed about COVID-19, and that this did not vary with education level, gender, or age. However, in their study, women more often reported feeling confused by the variety of information on COVID-19 than men, while no differences by education level were observed. Furthermore, the authors observed differences by education level depending on the type of media. Specifically, individuals with a low level of education more often reported that it is difficult to find information on the internet, but not in newspapers, magazines or on TV. No differences by gender were observed [69]. Further research is required to elucidate the relationships between assessment of perceived media coverage about COVID-19, education level, and risk perception in terms of protective behaviour.

Regarding compliance with recommended practices to combat the COVID-19 pandemic, a clear picture was not provided by the current results. Rather, the results varied with the type of behaviour, as well as between genders. In the bivariate analysis, highly educated women more often adhered to hand washing, and highly educated men more often adhered to maintaining distance compared with women and men with low levels of education, respectively. For mask wearing, however, no difference by education level was evident. These results are in accord with findings reported by Lüdecke and von dem Knesebeck [53], who also found educational differences in Germany with regard to hand washing and distance maintaining, but not mask wearing. However, they only included the initial phase of the pandemic and did not differentiate according to gender. In this context, it is also important to note that Ciancio et al. [48] found education-related differences in social distancing only among older people, but not in younger people. If this was also the case in Germany, differences according to education level may have been underestimated in the present analysis, in which people above the age of 75 were excluded.

However, when risk perception and media coverage were considered as well as knowledge in the multivariate models, women with low levels of education were found to be more likely to adhere to mask wearing, and men with low levels of education were found to be more likely to adhere to hand washing than highly educated women and men, respectively. We presume that this finding was likely due to education-related differences in knowledge and risk perception; the results of the moderation analyses revealed that–particularly in men–the impact of education level on protective behaviour varied with factual knowledge. Thus, with adequate factual knowledge, no educational differences in any protective behaviours were evident among men. However, when factual knowledge was erroneous, differences in education level were observed. This finding indicates that, in a situation where the low-education group had the same level of knowledge and risk perception as the high-education group, they would be likely to adhere to safety measures as often as the high-education group. Regarding severity, we found a similar result; when the disease was assessed as severe, there was no difference in education level, but when the disease was assessed as harmless, individuals in the low-educated group were less likely to maintain distance than the high-educated. Kim and Kim [70] reported a similar result in Korea, finding no effect of education level on protective behaviour when perceived severity was high. However, when perceived severity was low, a higher level of education was associated with more protective behaviour and a lower level of education was associated with less protective behaviour. The results could be interpreted by assuming that factual knowledge as well as risk communication about disease severity appeared to increase the chances of successfully implementing protective measures.

The current finding of only minor differences in protective behaviour according to education level might also be explained by the fact that contact restrictions and the wearing of a mask were officially ordered, and financial penalties were imposed for non-compliance.

Overall, the impact of education on risk perception, perceived media coverage, knowledge and protective behaviour was relatively small (e.g., a maximum of 0.26 units on a scale of 1–7 for risk perception). Education is only one social determinant alongside others, and we assume that many unobserved factors like household type (living alone or together with a partner and/or children) as well as income situation (which, for instance, can have an impact on owning a car or having to rely on public transport) and employment situation (e.g., the opportunity to work from home) may have further impact on risk perception, media perception, knowledge, and compliance with recommended protective practices [38, 42, 53]. Furthermore, gender and age also played an important role [23, 48, 52, 53, 71].

Our trend analysis revealed that both risk perception and protective behaviour were subject to substantial changes over time. We assume that the development of infection rates and the respective media coverage, as well as policy and communication regarding protective measures over time, played an important role. However, the trend analysis presented here remains merely descriptive. Further studies should analyse changes over the course of the pandemic in more detail. For example, more comprehensive time series analyses could produce valuable findings, as the long-lasting load caused by the pandemic requires the population to exhibit behavioural and mental adaptation to the situation. Analysing these adaptation processes and the role of education and health literacy within these may provide important insights. However, panel data would be a necessary requirement in order to observe intraindividual changes in cognitive and affective risk perception and their consequences on protective behaviour. To the best of our knowledge, no such data are available for Germany.

### Strengths and limitations

The main limitation of the present analysis is that the respondents’ education level only considered school leaving certificates, and not vocational qualifications. Because of this limited measure, we speculate that educational differences may have been considerably underestimated in the current study. If more fine-grained and precise data on educational certificates (particularly for higher level education such as university degrees) were available, greater educational differences would be likely to have appeared. This caveat, in turn, justifies the reporting and discussion of significant differences, even though some of them were relatively small. Additionally, the robustness of several educational differences was confirmed by their consistency over the course of the pandemic.

School leaving certificates are highly correlated with age, because younger birth cohorts tend to have more opportunities to obtain certificates that enable access to university, and thus have higher education levels. Therefore, only age-adjusted results were reported in the current study.

Another restriction is that people aged 75 and over, who belong to the main risk group, were not included in the current study. Because of the specific survey mode (online panel), and because respondents with lower levels of education are not part of the stratified sampling procedure in COSMO, inference of the results to the wider German population is limited. However, because age and sex (crossed) as well as federal state (uncrossed) were distributed according to the German population aged 18 to 75, differences between education levels within the COSMO sample provide a valid basis for evaluating educational differences in risk perception in Germany. Unfortunately, it was not possible to control for income, migration background or household type over the whole timespan, as these variables were not collected in all waves of the study.

When interpreting the total effects, it should also be acknowledged that, from June to the end of October (when infection rates were low) data were only collected biweekly. Therefore, the data included in the analyses over-represents periods with high incidence rates.

Despite the limitations mentioned above, the current study has some strengths. First, the study depicted the entire course of the pandemic in Germany to date, and, due to the ongoing survey, enables detailed descriptions of trends, starting at the very beginning of the pandemic. Moreover, the comparatively large sample size of the study allows for differentiated analyses.

## Conclusions

Risk perception and knowledge are key factors in the containment of the COVID-19 pandemic, impacting the adoption of protective behaviour [45, 52]. The current results indicate that education level should be considered in the development of risk communication strategies. A special focus should be given to increasing knowledge in groups with lower education levels using target group-specific communication strategies [72] including social media due to the COVID-19 “infodemic” [73] as well as the interaction of affect and knowledge [74].

Regarding our result that differences by education level were most pronounced in knowledge, the communication of protective behaviour should consider the heterogeneity of individuals’ ability to understand and evaluate pandemic-related information. Achieving a better understanding of these kinds of health information suggests that the promotion of health literacy appears to be a promising approach [75, 76]. Knowledge is one part of the cognitive dimensions of health literacy [77]. COVID-19-related knowledge not only helps people understand which protective behaviour should be applied, but can also influence the motivation to comply [75]. However, promoting health literacy should not be restricted to providing factual knowledge, but should also help people assess the validity of information and understand that information can change based on new scientific findings [76, 78, 79]. At the same time, authorities should address uncertainties in knowledge transparently [80] to disprove irrational fears, misinformation and rumours [72, 81], as well as addressing emotional concerns and worries [81]. To achieve these targets of risk communication in the COVID-19 pandemic by considering educational levels, public authorities should involve stakeholders of target groups with different educational levels in developing appropriate risk communication strategies.
